# Seeking bridge symptoms of anxiety, depression, and sleep disturbance among the elderly during the lockdown of the COVID-19 pandemic—A network approach

**DOI:** 10.3389/fpsyt.2022.919251

**Published:** 2022-08-03

**Authors:** Liang Zhang, Yanqiang Tao, Wenxin Hou, Haiqun Niu, Zijuan Ma, Zeqing Zheng, Shujian Wang, Shuang Zhang, Yichao Lv, Qiubai Li, Xiangping Liu

**Affiliations:** ^1^College Students' Mental Health Education Center, Northeast Agricultural University, Harbin, China; ^2^College of Education for the Future, Beijing Normal University at Zhuhai, Zhuhai, China; ^3^Beijing Key Laboratory of Applied Experimental Psychology, Faculty of Psychology, Beijing Normal University, Beijing, China; ^4^School of Psychology, Nanjing Normal University, Nanjing, China; ^5^School of Psychology, South China Normal University, Guangzhou, China; ^6^State Key Laboratory of Cognitive Neuroscience and Learning, Beijing Normal University, Beijing, China; ^7^Political and Legal Committee of Xiangfang District, Harbin, China

**Keywords:** depression, anxiety, sleep disturbance, elderly, network

## Abstract

**Background:**

Besides physical changes, elderly adults are prone to have mental disorders such as anxiety, depression, and sleep disturbance, and the pandemic of COVID-19 worsened the situation. However, internal relationships and co-occurrence of psychopathologies were scarcely examined. Therefore, in the current study, through network analysis, we inspected relationships among symptoms of depression, anxiety, and sleep disturbance and identified key symptoms that espoused the disease.

**Methods:**

We asked 1,302 elderly adults to fill in Patient Health Questionnaire-2 (depressive symptoms), the Generalized Anxiety Disorder-2 (anxiety symptoms), and the Youth Self-rating Insomnia Scale (sleep disturbance) and then constructed three networks for elderly adults, male elderly, and female elderly. *Via* network analysis, we accomplished four goals. First, we identified symptom with the highest centrality (i.e., strength) index for each network; then, we found the strongest correlation (i.e., edges) in each network; thirdly, we confirmed specific nodes that could bridge anxiety, depression, and sleep disturbance; the last was to compare networks based on genders. Network stability and accuracy tests were performed.

**Results:**

Networks of elderly adults, male elderly, and female elderly were stable, accurate, and intelligible. Among all networks, “Nervousness”- “Excessive worry” (GAD-1- GAD-2) had the strongest correlation, and “Nervousness” (GAD-1) had the highest strength and bridge strength value. When we made a comparison between female elderly's and male elderly's networks, except for the significant difference in the mean value of “Difficulty initiating sleep” (YSIS-3), the findings showed that the two networks were similar. Network stability and accuracy proved to be reliable.

**Conclusions:**

In networks of anxiety, depression, and sleep disturbance, anxiety played a conspicuous role in comorbidity, which could be a target for practical intervention and prevention.

## Introduction

In China, the aging population occupies 17.88% of the total demographic composition (the cut-off age is 60) (1). Getting aged can bring both cognitive and emotional changes. Previous studies proved that aging-induced gut microbiota compositions were the main causes of cognitive decline (2), and elderly adults were impressionable to mental disorders, such as depression (3). Physical and mental illness perturbed elderly adults' routine life and impaired their behaviors and cognitive capabilities. However, compared to other populations, studies into the elderly' mental health were rare. Thus, attention to the aging population is necessary for alleviating the national economic burden and helping aged population improve a sound life.

However, the COVID-19 is a challenge for elderly adults, with an infection rate of 25.3% (4). As well as the general population which increased smoking, video playing and drinking frequency (5) and health care workers who were beset by suicidal ideation (6), elderly adults were victims of the COVID-19 suffering from anxiety, depression and poor sleep quality (7, 8). Moreover, due to city lockdown, diagnosis of mental disorders or cognitive function decline was delayed (9). Hence, probing into elderly adults' mental health is necessary for early diagnosis and intervention.

Among negative mental and physical problems, sleep disturbance such as nocturnal and earlier waking are widespread and striking (10). Generally, female elderly report more sleep disturbance (11). Malnutrition, lack of exercise, long-time TV watching, and illness can be stressors of sleep disturbance (12). Superficially, sleep disturbance hinders elderly adults from performing daily roles such as taking care of children or driving. However, sleep disturbance can damage physical health by causing slips/falls (13) or dizziness and impair cognitive function (14). Moreover, sleep disturbance is positively related to mental disorders such as depression and anxiety (15), though few studies were done to correlations between sleep disturbance and mental health for the aged population. On the background of city lockdown, delving into the relationship between sleep disturbance, anxiety, and depression is meaningful for elderly adults in both diagnosis and intervention.

Major depression, consisting of symptoms including anhedonia, hopelessness, losing appetite, and abnormal weight changes, has a growing trend in the aged population and is more prevalent in female elderly (3). Moreover, as a common comorbidity of depression, though exclusive studies on the anxiety of elderly adults are scarce, anxiety alone is another deleterious psychological burden for elderly adults and is more common in female elderly (16). There is a body of studies stressing external risk factors such as a pandemic that can elicit depression or anxiety, whilst few have done into the concurrence of sleep disturbance, depression, and anxiety, especially in the aged population, even sleep disturbance is strongly correlated to psychopathologies (17). Moreover, previous studies mainly emphasized the unidimensional causal relationship that treatment of depression or anxiety demonstrated an alleviation in sleep quality (18) while ignored that sleep quality improvement can espouse the rehabilitation of depression and anxiety. In the current study, we aimed to reveal the bidirectional relationship between sleep disturbance, anxiety, and depression, in which poor sleep quality can cause anxiety or depression while anxiety or depression plays a reactive role in enfeebling sleep quality.

However, traditional statistical methods cannot reveal bidirectional relationships since traditional theories such as the latent approach hold the view that all visible variables are independent and loosely allied to present latent variables (19). However, symptoms such as waking up earlier, nervousness, or feeling depressed are dynamically interwoven with each other in both mental disease diagnosis and treatment. In other words, improvement or degeneration of either waking up earlier, excessive worry, feeling depressed, or other visible symptoms could inevitably cause changes in the whole symptom structure. In order to manifest latent variables as well as interactions among symptoms, in the current study, we applied a newly proposed method, network analysis, to reveal the complex interactions among symptoms composing sleep disturbance, depression, and anxiety (20). According to network analysis, complicated psychopathology emerges from the interactions among visible variables (21). Therefore, different from a simple combination of symptoms, it can be that elderly adults trap in a depressive mood for spasmodic waking up earlier and during the night, being unable to fall asleep deepens their depressive mood. Moreover, network analysis provides us with a new perspective to evaluate how symptoms (nodes) function and how symptoms interweave with each other (edges) (22). Hence, we can identify consequential symptoms and relationships to guide more effective treatment and interventions.

As a short summary, in the current study, we asked 1,302 elderly adults to obtain a straightforward view of both symptoms of depression, anxiety, and sleep and symptoms' interactions, targeting to clarify critical issues in intervention and prevention.

## Methods

### Participants

We recruited 1,302 (male = 409, female = 893) participants from Harbin, Heilongjiang Province, to fill in questionnaires posted on Wenjuanxing (https://www.wjx.cn), an online questionnaire platform. All participants signed electronic informed consent before the assessment. Ethical committee of the ^***^ university approved this study (Reference number: 202112220084).

In participants selection, we obeyed the following criteria: (1): over 60 years old, without gender restriction; (2): attended the test with informed consent; (3): stayed in Harbin during 12^th^ November 2021 to 15^th^ November,2021, the period of city lockdown. Participants were excluded from the current study: (1): participants' health conditions might diminish for a lethal disease including heart, lung, brain, and other critical diseases; (2): usage of anti-anxiety or anti-depression medications; (3): refused or could not complete the questionnaires; (4): failed to provide completely informed consent owing to cognitive or behavioral disability; (5) in clinical trials of other psychopathological drugs; (6): did not stay in Harbin from 12^th^ November, 2021 to 15^th^ November,2021.

### Measurements

#### Generalized Anxiety Disorder- 2 (GAD-2)

The Generalized Anxiety Disorder Scale (GAD-2) is a valid and reliable assessment to screen generalized anxiety symptoms (23). The Chinese version also has good psychometric properties for identifying anxiety (24). Participants answered two questions about the frequency of core anxiety symptoms over the last 2 weeks. Each item scores from 0 (not at all) to 3 (nearly every day) (25). Higher scores indicate more severe anxiety propensity. In the current study, GAD-2 had high Cronbach α values of 0.89, 0.93, and 0.91 for the elderly, male elderly, and female elderly groups, respectively.

#### Patient Health Questionnaire (PHQ-2)

The two-item Patient Health Questionnaire (PHQ-2) is widely used in screening for depressive symptoms (26). All participants were asked about the frequency 0 (not at all) to 3 (nearly every day) of experiencing given depressive symptoms in the last 2 weeks and higher scores indicate more severe depressive symptoms. The Chinese version of PHQ-2 has been proved to be valid and reliable (27), and in the current study, PHQ-2 has high Cronbach α values of 0.84, 0.84, and 0.89 in the elderly adults, male elderly and female elderly groups respectively.

#### Youth Self-rating Insomnia Scale (YSIS-3)

In the current study, we selected 3 questions from YSIS-8 (28), a 5-point Likert questionnaire assessing sleep disturbance in the last month. Participants answered 3 questions about “Difficulty initiating sleep,” “Difficulty maintaining sleep” and “Early morning awakening” scoring from 1 (Very Satisfied) to 5 (Very Unsatisfied). Total scores in this questionnaire ranged from 3 to 15. Higher scores indicated poorer sleep quality. The previous study proved YSIS-3 in Chinese to be valid and reliable (29). Cronbach α values of 0.93, 0.94, and 0.93 indicated a high internal consistency of YSIS in the current study.

### Statistical analysis

#### Item check

All analyses were done by R (Version 4.1.2). *DescrTable* in R-package *compareGroups* was used to check item informativeness. Mean value, standard deviation (*SD*), kurtosis, skewness of items, and polychoric correlations were assessed first. Item informativeness was assessed by the mean value of standard deviation [i.e., ± 2.5 standard deviations (*SD*) around mean standard deviation (*SD*)] (30). In other words, an informative item should have a value in the range of ± 2.5 standard deviations (*SD*) around the mean item's standard deviation (*SD*), otherwise, the item was non-informative.

In network estimation, an Extended Bayesian Information Criterion (EBIC) model with the least absolute shrinkage and selection operator (LASSO) was used to establish networks (31). Partial correlation analysis, keeping all other variables constant, was computed to indicate the association of each pairwise variables and form networks. Moreover, to get a sparse and intelligible network, LASSO and EBIC were chosen to remove spurious correlations (32). In network analysis, nodes represent symptoms or variables, and edges represent partial correlation coefficients between two nodes (33). Higher correlations are shown in thicker and more saturated edges. Positive and negative correlations are shown in blue and red, respectively (34). In the part of estimation and visualization, we applied the R-packages *qgraph 1.9* and *bootnet 1.5* (31, 34).

In psychological network analysis, strength (i.e., the sum value of all absolute edge weights between one specific node and other nodes connected to it.) is the reliable and necessary centrality index that must be computed (35). Closeness (i.e., the inverse of the sum value of distances between one node and all other nodes in the network) or betweenness (i.e., fractions of short paths pass one specific node) are not suitable to measure nodes' importance in psychological networks (35).

#### Network stability and accuracy

We used an R package *bootnet* (version 1.5) (31) to test networks' stability and accuracy. First, we applied non-parametric bootstrapping to test the edge weights accuracy with 95% bootstrap *CI*s (36). In the previous study, large edge weights *CI*s indicated poor accuracy of edges (37). Then we investigated centrality stability with case-dropping subset bootstrap by measuring the correlation stability coefficient (*CS-coefficient*), which represents the maximum proportion of dataset that can be removed when two data sets maintain the association above the 0.7 level with a 95% confidence interval (33). A previous study recommended a *CS-coefficient* value should be preferably above 0.5 and should not be lower than 0.25 (38). Besides testing edge weights accuracy and centrality stability, we also tested whether there were significant differences between edges and nodes using the *bootstrap difference test*. In this step, the null-hypothesis test was checked to see if zero existed in the bootstrapped *CI*s (38).

In addition, to test network stability and predictability, a metric quantifies how well one node can be estimated by all its neighboring nodes (39), was estimated by *mgm* (Version 1.2-12) in R (39). High predictability indicates strong mutual interactions in the network and vice versa (40). Bridge symptoms are represented by one or more nodes that can strengthen interactions among mental disorders (41) by *bridge* function in the R package *networktools* (Version 1.4.2) (42). In the current study, the bridge symptoms centrality was represented by bridge strength.

#### Comparison of the network structure between genders

To compare two networks (i.e., elderly male and elderly female), we applied a permutation test, the Network Comparison Test (NCT), which was conducted with 1,000 permutations through “*NetworkComparisonTest*” (43). First, we tested the null hypothesis that there was no statistically significant difference between the two networks' global strengths (i.e., the absolute sum of all node strength) (44). Besides, we tested the null hypothesis that all edge weights in two networks did not differ significantly. Moreover, we tested the variance of individual edge weights in two networks using the Holm-Bonferroni value of 0.05.

## Results

### Descriptive statistics and item check

First, items' informativeness (i.e., standard deviation (SD), mean value, kurtosis, skewness) was computed and shown in Table 1. We found no items were poorly informative (i.e., 2.5 *SD* below or above the mean level of items' *SD*).

**Table 1:** Statistical description.

**Group**	**Variables**	**Label**	***N***	***Mean***	***SD***	***Skew***	***Kurtosis***	***Predictability*** ***(R^2^)***
Elderly	GAD-1	Nervousness	1,301	0.50	0.84	1.67	1.88	0.65
	GAD-2	Uncontrollable worry	1,301	0.41	0.74	1.96	3.33	0.69
	PHQ-1	Anhedonia	1,301	1.40	0.73	1.93	3.26	0.77
	PHQ-2	Sad mood	1,301	1.37	0.74	2.09	3.71	0.75
	YSIS-3	DIS	1,301	1.76	1.13	1.46	1.18	0.72
	YSIS-4	DMS	1,301	1.88	1.21	1.25	0.47	0.77
	YSIS-5	EMA	1,301	1.83	1.19	1.37	0.80	0.77
Male	GAD-1	Nervousness	409	0.53	0.86	1.60	1.60	0.61
	GAD-2	Uncontrollable worry	409	0.41	0.75	1.95	3.29	0.68
	PHQ-1	Anhedonia	409	1.42	0.73	1.86	2.97	0.79
	PHQ-2	Sad mood	409	1.39	0.73	1.95	3.17	0.78
	YSIS-3	DIS	409	1.67	1.09	1.75	2.26	0.76
	YSIS-4	DMS	409	1.83	1.20	1.37	0.81	0.79
	YSIS-5	EMA	409	1.75	1.13	1.52	1.42	0.80
Female	GAD-1	Nervousness	892	0.49	0.83	1.70	2.01	0.68
	GAD-2	Uncontrollable worry	892	0.41	0.74	1.95	3.33	0.70
	PHQ-1	Anhedonia	892	1.39	0.73	1.97	3.40	0.76
	PHQ-2	Sad mood	892	1.37	0.74	2.14	3.93	0.74
	YSIS-3	DIS	892	1.80	1.14	1.34	0.78	0.71
	YSIS-4	DMS	892	1.90	1.21	1.19	0.32	0.76
	YSIS-5	EMA	892	1.87	1.22	1.30	0.56	0.76

### Network structure and centrality measures analysis

Three raw networks of the elderly, male elderly, and female elderly were shown in Supplementary Figure S1, respectively, and the partial correlation matrices were enclosed in Supplementary Tables 1–3.

#### Elderly network

**Figure 1:**
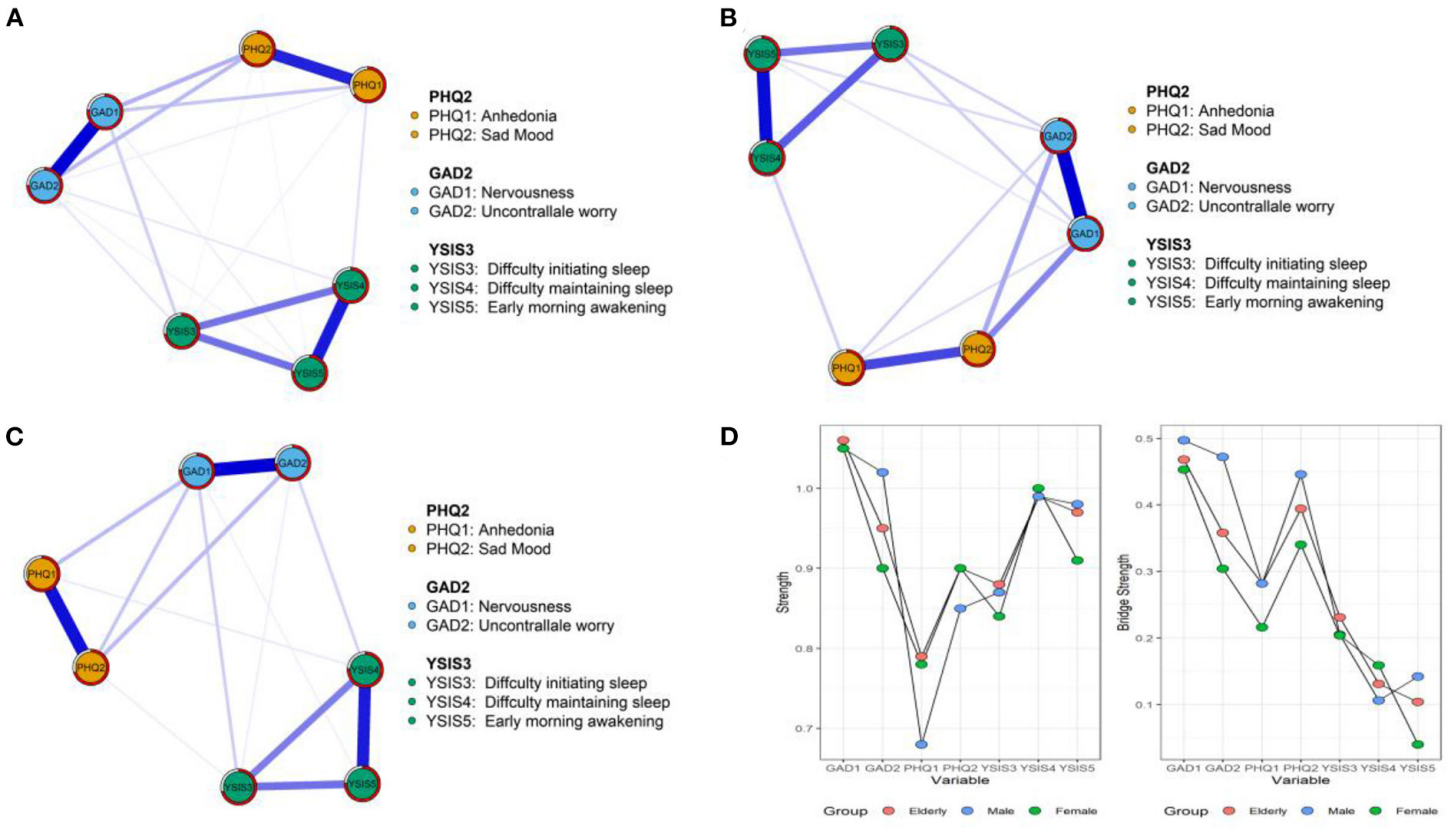
The network structures of anxiety, depressive symptoms, and sleep quality. **(A)** All elderly. **(B)** Male elderly. **(C)** Female elderly. **(D)** Strength and bridge strength values.

In the network with predictability (i.e., *R*^2^) of all elderly adults (Figure 1A), the edge of “Nervousness”- “Uncontrollable worry” (GAD-1-GAD-2) had the strongest correlation, followed by the edge of “Difficulty maintaining sleep”— “Early morning awakening” (YSIS-4-YSIS-5) and the edge of “Anhedonia”— “Sad mood” (PHQ-1-PHQ-2).

For centrality indices presented in Figure 1D, we reported strength and bridge strength for 3 networks, respectively. In the network of elderly adults, “Nervousness” (GAD-1) had the highest strength value, followed by “Early morning awakening” (YSIS-5) and “Uncontrollable worry” (GAD-2). In the part of bridge strength, “Nervousness” (GAD-1), “Sad mood” (PHQ-2), and “Uncontrollable worry” (GAD-2) had the highest bridge strength values.

#### Male elderly network

In the network of male elderly (Figure 1B), most weighted edges were the same as those in the elderly adults' network. From centrality indices, “Nervousness” (GAD-1), “Uncontrollable worry” (GAD-2), and “Difficulty maintaining sleep” (YSIS-4) had the highest strength values. As for bridge strength shown in Figure 1D, “Nervousness” (GAD-1), “Uncontrollable worry” (GAD-2), and “Sad mood” (PHQ-2) had the highest centrality values.

#### Female elderly network

In the network of female elderly (Figure 1C), “Nervousness”— “Uncontrollable worry” (GAD-1-GAD-2), “Difficulty maintaining sleep”- “Early morning awakening” (YSIS-4-YSIS-5) and “Anhedonia”- “Sad mood” (PHQ-1-PHQ-2) had the strongest correlations. As for node strength, “Nervousness” (GAD-1) had the most strength, followed by “Difficulty maintaining sleep” (YSIS-4) and “Early morning awakening” (YSIS-5). As bridge strength presented in Figure 1D, nodes of “Nervousness” (GAD-1), “Sad mood” (PHQ-2), and “Uncontrollable worry” (GAD-2) had the highest centrality values, which were the same as the elderly adults' network.

### Network accuracy and stability

Predictability values of the three networks were 0.76 (*M*_*predictability*_ = *0.73* ± *0.06*), 0.80 (*M*_*predictability*_ = *0.75* ± *0.07*), and 0.74 (*M*_*predictability*_ = *0.73* ± *0.03*), indicating that one specific node can be predicted or explained by its neighboring nodes at the rate over 73% (Table 1).

In the edge weights accuracy test (Supplementary Figure S2), bootstrapped *CI*s were narrow, indicating that edges in three networks were reliable. In addition to edge weights accuracy and case-dropping bootstrap, in the non-parametric bootstrap procedure, edge weights and node centrality indices differed statistically significant, as shown in Supplementary Figures 3, 4.

**Figure 2:**
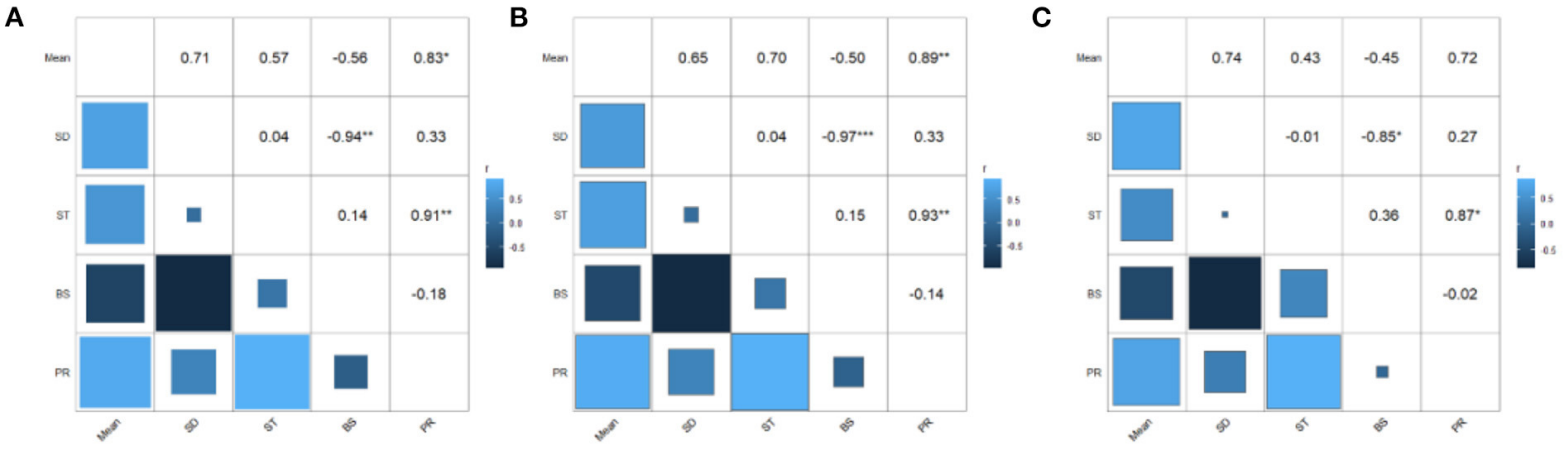
The matrix of correlations between mean value, standard deviation, strength, bridge strength, and predictability. **(A)** Elderly adults. **(B)** Male elderly. **(C)** Female elderly.

Moreover, in the network of elderly adults, male elderly and female elderly, strength and predictability [*r*_*s*_ = 0.91^**^ (0.51; 0.99); *r*_*s*_ = 0.93^**^ (0.61; 0.99); *r*_*s*_ = 0.87^*^ (0.34; 0.98)] were significantly related with each other, indicating that predictability was reliable. Besides, in the network of the elderly adults, there were significant correlations between *SD* and predictability [*r*_*s*_ = 0.83^**^ (0.21; 0.97)] and between strength and predictability [*r*_*s*_ = −0.94^**^ (-0.99;−0.63)]. In the network of male elderly, mean value and predictability correlated at a significant level [*r*_*s*_= 0.89^**^ (0.40; 0.98)] so as *SD* and bridge strength [*r*_*s*_ = −0.97^***^ (-1.00;−0.82)]. In the network of female elderly, *SD* and bridge strength [*r*_*s*_ = −0.85^**^ (-0.98;−0.29)] correlated at a significant level (Figure 2).

### Networks comparison between two sexes

**Figure 3:**
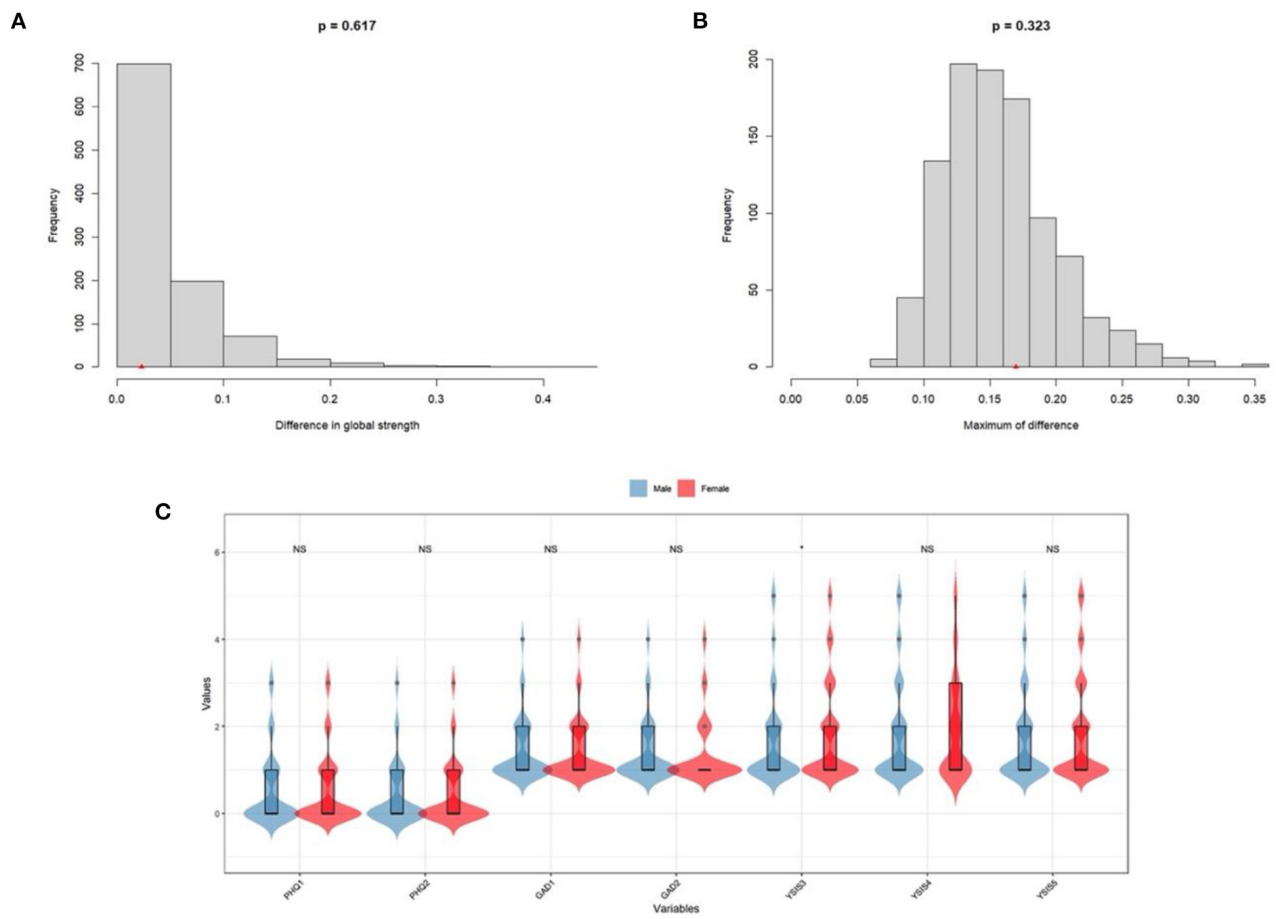
Network comparison between male elderly and female elderly. **(A)** Global strength. **(B)** Edge weights. **(C)** Mean scores on items between male elderly and female elderly.

As mentioned above, we investigated differences between the male elderly network and female elderly network through Network Comparison Test (NCT). First, as shown in Figure 3A, we tested the strength difference of the two sexes through NCT (male = 3.36, female = 3.29, *S* = 0.02, *p* = *0.62*). Then in Figure 3B, from the perspective of the edge weights (1,000 permutations) test, the results showed no significant difference among edge weights in two networks (*M* = *0.17, p* = *0.32*). In Figure 3C, from *t*-test between mean item scores, we found that there were no significant differences in items' mean value (*p* > *0.05*) except for item “Difficulty initiating sleep” (YSIS-3) (*p* < *0.05*).

## Discussion

In the current study, we asked 1,302 elderly adults to fill in three reliable questionnaires and analyzed the symptom structures of sleep disturbance, anxiety, and depression. Several results are worth discussing.

Our findings indicated that in networks of elderly adults, male elderly and female elderly, “Nervousness” - “Uncontrollable worry” (GAD-1- GAD-2) had the strongest association which meant that on symptom level, Nervousness (GAD-1) and Uncontrollable worry (GAD-2) interplayed in engendering anxiety. The current results proved that compared to traditional statistical approaches, network analysis could better reveal intrinsic interactions between observable variables. Uncontrollable worry is a cognitive phenomenon related to repetitive concerns or thinking about potential negative events, hazards, or risks (45), and nervousness is an uneasy feeling to cope with imminent disasters. Due to the impact of filial piety in Confucius's concept (46), in a typical Chinese family, elderly adults are regarded as the leaders in making decisions and cultivating younger members. Meanwhile, younger members should venerate and care for elderly members. Thus, Chinese elderly adults are particularly worried about illness or poverty from which they may fail to support and lead the family or become a heavy burden to their children for physical illness and low social-economic status (47). In this reactive chain, elderly adults' great concerns for the family can strengthen their nervousness about imminent issues. Besides medication treatment, mindfulness therapy (MT), acceptance and commitment therapy (ACT), moving to emptiness (MET) and cognitive-behavioral therapy (CBT) are effective in anxiety mitigation (48, 49).

The strong correlation of “Difficulty maintaining sleep”—“Early morning awakening” (YSIS-4-YSIS-5) across three networks clarified that observable sleep complaint factors mutually interact. “Difficulty maintaining sleep” is measured by increased wake time, frequency of arousals, and periodic limb movements during sleep (50). Due to age-related changes in circadian rhythm timing and behavioral changes, elderly adults cannot maintain deep sleep for a long time (10). Stress, alcohol consumption, lack of exercise (51), and lack of social support (52) can further impair sleep quality. We evinced the association between YSIS-4 and YSIS-5 was that elderly adults could not keep asleep for their stress or illness. Thus, they left their beds earlier, seeking for relief or treatment. The experience of waking up earlier perturbed elderly adults' sleep quality through long-time day naps, and elderly adults failed to sleep well during the night. Pharmacological interventions display meaningful outcomes with better sleep patterns (53).

We have found that “Anhedonia”- “Sad mood” (PHQ-1-PHQ-2) had a strong connection which implied that elderly adults lost interest in the time of feeling depressed, and in the depressive mood, elderly adults felt like doing nothing. Lack of social support, chronic diseases, and substance abuse are risk factors for depression (3). Compared to youths, elderly adults are more prone to be anhedonia virtually of less curiosity. In the status of anhedonia, elderly adults attribute internally that they are bootless, the root of depression or sad mood. It is a reactive chain that elderly adults' depression can deepen anhedonia. In treatment, psychotherapy (54) or even physical activities (55) can effectively ameliorate symptoms.

Though in the network comparison, networks between male elderly and female elderly did not differ significantly in global strength or edge weights, variances in node strength and bridge strength are worthy of discussion. In both networks of the male and female elderly, “Nervousness” (GAD-1) had the most centrality strength and bridge strength value, indicating that anxiety was the prominent symptom in anxiety, depression, and sleep disturbance manifestation and linkage. Furthermore, “Uncontrollable worry” (GAD-2) had the second strongest node strength and bridge strength in the network of male elderly, suggesting that anxiety symptoms were outstanding and critical in releasing male elderly's anxiety, depression, and sleep disturbance. We boldly infer that in Chinese families, male elderly manage family expenditure and income. As a result, male elderly tend to exhibit high levels of anxiety in the confrontation of less income (e.g., city lockdown) and physical illness, and without intervention, anxiety symptoms can drag male elderly into depression or sleep disturbance.

However, compared to male elderly, females demonstrated more sleep disturbance in later life since in the network, “Difficulty maintaining sleep” and “Early morning awakening” (YSIS-4 &YSIS-5) had the second and third highest node strength. Our finding was consistent with a previous study, which found that females suffered more from sleep problems than males (11). In China, females are required to undertake housework. The COVID-19 has profound physical and psychological impacts on female elderly. Higher standard in sanity, more familial member to be cared and less space for leisure activities can be regarded as stressor of sleep disturbance.

## Limitation

As if using GAD-2, PHQ-2 and YSIS-3 were convenient for elderly adults to fill in forms in a short time, seven questions could not comprehensively reveal symptoms structures. In further studies, more items should be contained. Moreover, to delineate the developmental process of anxiety, depression, and sleep disturbance, longitude studies should be done. Demographical information on occupation, marital status, educational level, and socioeconomic status should also be counted in further investigation.

## Conclusion

In the current study, we included anxiety, depression, and sleep disturbance to construct three networks of elderly adults, male elderly and female elderly, respectively. The results showed that “Nervousness” and “Uncontrollable worry” (GAD-1- GAD-2) had the strongest correlation implying that network analysis, this newly proposed methodology, could present ingenuous relationships in variables. Moreover, clinically, key symptoms and symptoms links were pointed out for intervention and diagnosis so that clinicians can alleviate symptoms within less time and with greater treatment outcomes.

## Data availability statement

The raw data supporting the conclusions of this article will be made available by the authors, without undue reservation.

## Ethics statement

The studies involving human participants were reviewed and approved by Faculty of Psychology, Beijing Normal University (Reference Number: 202112220084). The patients/participants provided their written informed consent to participate in this study.

## Author contributions

Study design: XL. Data collection: LZ and QL. Analysis and interpretation: LZ, YT, and WH. Drafting of the manuscript: LZ. Critical revision of the manuscript: ZM, WH, HN, SW, YL, ZZ, and SZ. All authors contributed to the article and approved the submitted version.

## Conflict of interest

The authors declare that the research was conducted in the absence of any commercial or financial relationships that could be construed as a potential conflict of interest.

## Publisher's note

All claims expressed in this article are solely those of the authors and do not necessarily represent those of their affiliated organizations, or those of the publisher, the editors and the reviewers. Any product that may be evaluated in this article, or claim that may be made by its manufacturer, is not guaranteed or endorsed by the publisher.
