# Supplementary material for: Seeking bridge symptoms of anxiety, depression, and sleep disturbance among the elderly during the lockdown of the COVID-19 pandemic—A network approach
Source: Front Psychiatry. 2022 Aug 3;13:919251. doi: 10.3389/fpsyt.2022.919251 (PMC9381922; doi:10.3389/fpsyt.2022.919251)
Supplement: Supplementary file 1 [file Data_Sheet_1.DOCX]

**Supplementary materials**

**Table S1.** Weighted adjacency matrix among ederly adults.

**Table S2.** Weighted adjacency matrix for male elderly.

**Table S3.** Weighted adjacency matrix for female elderly.

**Table S4.** Means, standard deviations, *t* test, *p value*, and *Cohen's d* in males (n = 409) and females (n = 893).

**Fig. S1.** Three raw networks of elderly adults, male elderly and female elderly.

**Fig. S2.** Nonparametric bootstrapped confidence intervals of estimated edges. The red line represents the estimated edge, while the shaded area indicates the 95% bootstrap confidence interval.

**Fig. S4.** Nonparametric bootstrapped difference test for edges. Grey boxes indicate no significant difference, whereas black boxes indicate a statistically significant difference (*p < .05*). Diagonal colour and saturation represent the magnitude and direction of each estimated edge.

**Fig. S5.** Nonparametric bootstrapped difference test for nodes. Grey boxes indicate no significant difference, whereas black boxes indicate a statistically significant difference (*p < .05*). Diagonal colour and saturation represent the magnitude and direction of each estimated node.

**Table S1. Weighted adjacency matrix among elderly adults (*p < .05*).**

|  | PHQ1 | PHQ2 | GAD1 | GAD2 | YSIS3 | YSIS4 | YSIS5 |
| --- | --- | --- | --- | --- | --- | --- | --- |
| PHQ1 | 0.00 | 0.51 | 0.13 | 0.05 | 0.03 | 0.07 | 0.00 |
| PHQ2 | 0.51 | 0.00 | 0.18 | 0.16 | 0.03 | 0.00 | 0.03 |
| GAD1 | 0.13 | 0.18 | 0.00 | 0.59 | 0.11 | 0.00 | 0.05 |
| GAD2 | 0.05 | 0.16 | 0.59 | 0.00 | 0.06 | 0.06 | 0.03 |
| YSIS3 | 0.03 | 0.03 | 0.11 | 0.06 | 0.00 | 0.32 | 0.33 |
| YSIS4 | 0.07 | 0.00 | 0.00 | 0.06 | 0.32 | 0.00 | 0.53 |
| YSIS5 | 0.00 | 0.03 | 0.05 | 0.03 | 0.33 | 0.53 | 0.00 |

|  | PHQ1 | PHQ2 | GAD1 | GAD2 | YSIS3 | YSIS4 | YSIS5 |
| --- | --- | --- | --- | --- | --- | --- | --- |
| PHQ1 | 0.00 | 0.40 | 0.08 | 0.10 | 0.00 | 0.11 | 0.00 |
| PHQ2 | 0.40 | 0.00 | 0.26 | 0.19 | 0.00 | 0.00 | 0.00 |
| GAD1 | 0.08 | 0.26 | 0.00 | 0.55 | 0.10 | 0.00 | 0.06 |
| GAD2 | 0.10 | 0.19 | 0.55 | 0.00 | 0.10 | 0.00 | 0.08 |
| YSIS3 | 0.00 | 0.00 | 0.10 | 0.10 | 0.00 | 0.36 | 0.31 |
| YSIS4 | 0.11 | 0.00 | 0.00 | 0.00 | 0.36 | 0.00 | 0.53 |
| YSIS5 | 0.00 | 0.00 | 0.06 | 0.08 | 0.31 | 0.53 | 0.00 |

**Table S2. Weighted adjacency matrix among male elderly (*p < .05*).**

**Table S3. Weighted adjacency matrix among female elderly (*p < .05*).**

|  | PHQ1 | PHQ2 | GAD1 | GAD2 | YSIS3 | YSIS4 | YSIS5 |
| --- | --- | --- | --- | --- | --- | --- | --- |
| PHQ1 | 0.00 | 0.56 | 0.16 | 0.00 | 0.00 | 0.05 | 0.00 |
| PHQ2 | 0.56 | 0.00 | 0.14 | 0.16 | 0.05 | 0.00 | 0.00 |
| GAD1 | 0.16 | 0.14 | 0.00 | 0.60 | 0.12 | 0.00 | 0.04 |
| GAD2 | 0.00 | 0.16 | 0.60 | 0.00 | 0.04 | 0.10 | 0.00 |
| YSIS3 | 0.00 | 0.05 | 0.12 | 0.04 | 0.00 | 0.30 | 0.34 |
| YSIS4 | 0.05 | 0.00 | 0.00 | 0.10 | 0.30 | 0.00 | 0.54 |
| YSIS5 | 0.00 | 0.00 | 0.04 | 0.00 | 0.34 | 0.54 | 0.00 |

**Fig. S1. Three raw networks of elderly adults, male elderly and female elderly.**


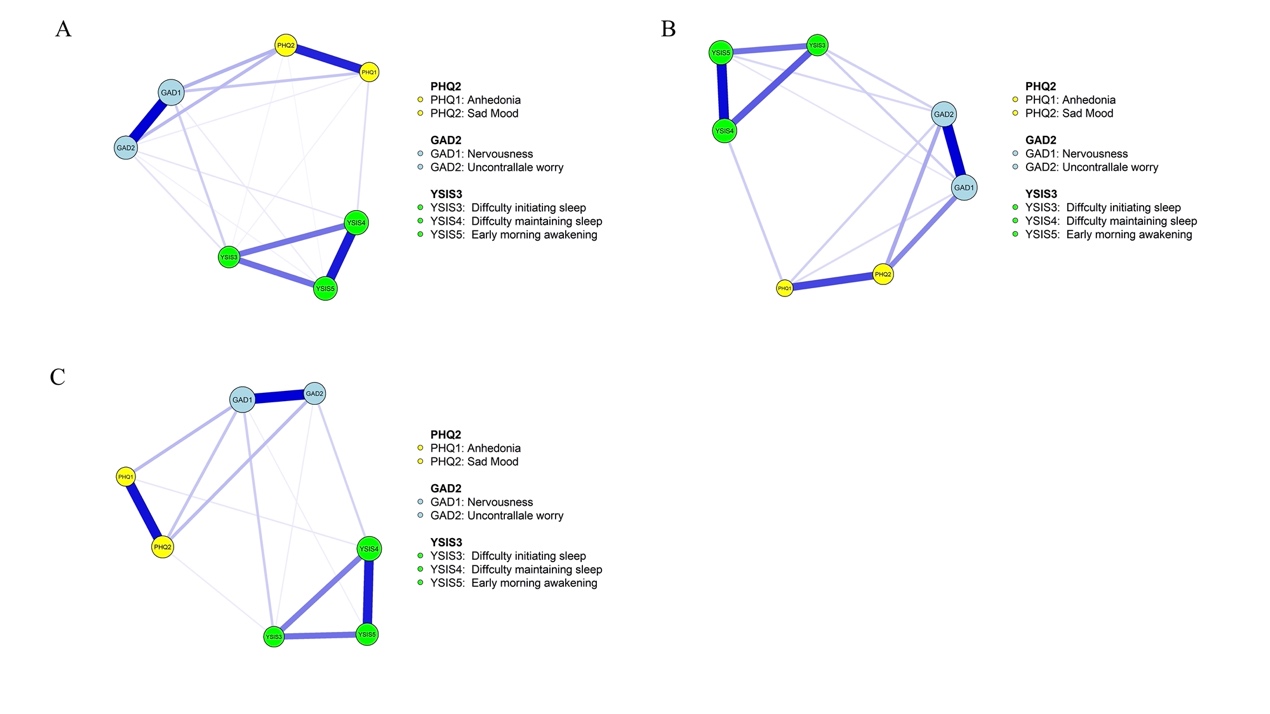


Fig. S1. The network structure of anxiety, depressive symptoms, and sleep complaints of A, elderly adults. B, male elderly. C, female elderly.

**
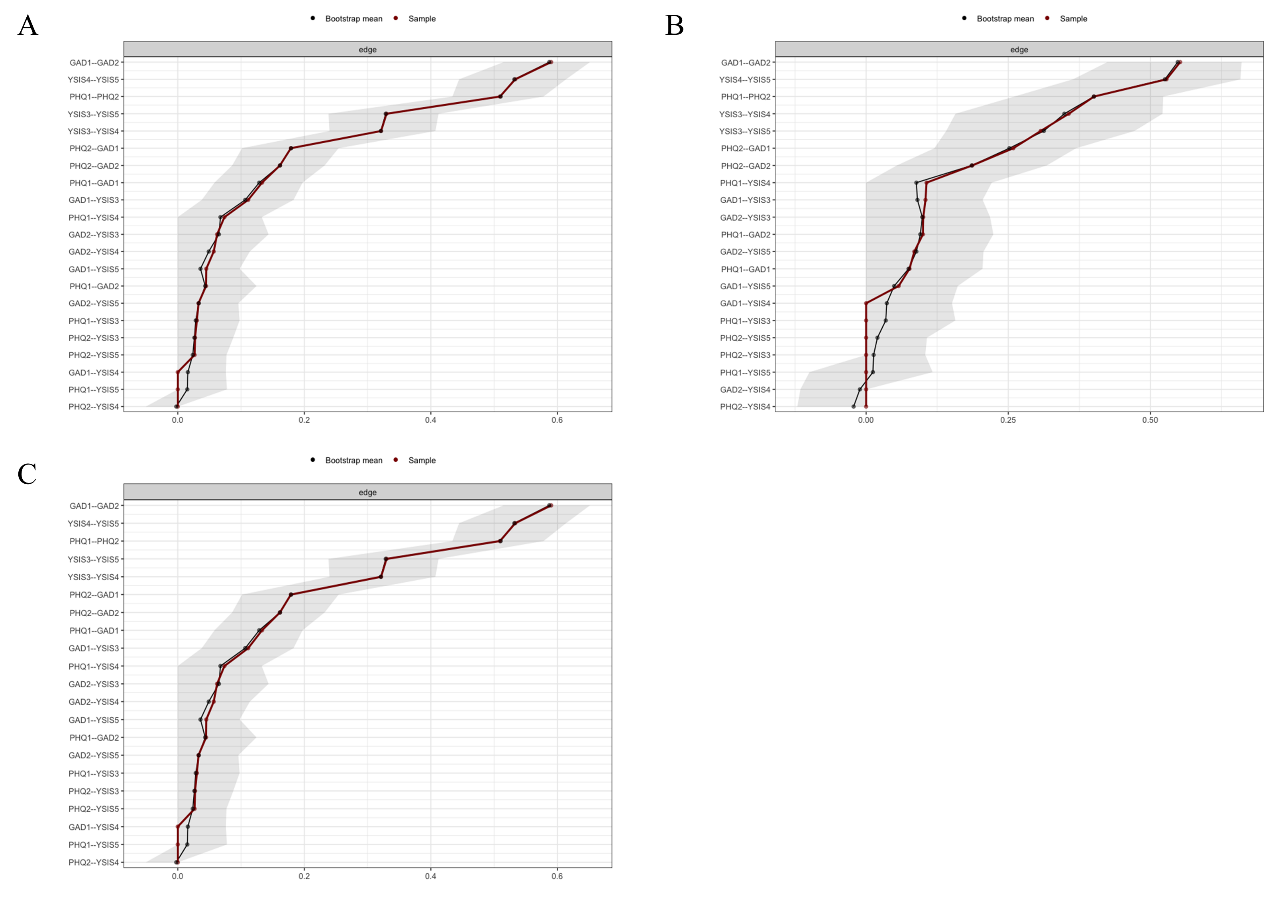
**

**Fig. S2.** Nonparametric bootstrapped confidence intervals of estimated edges. The red line represents the estimated edge, while the shaded area indicates the 95% bootstrap confidence interval, A, elderly adults. B, male elderly. C, female elderly.


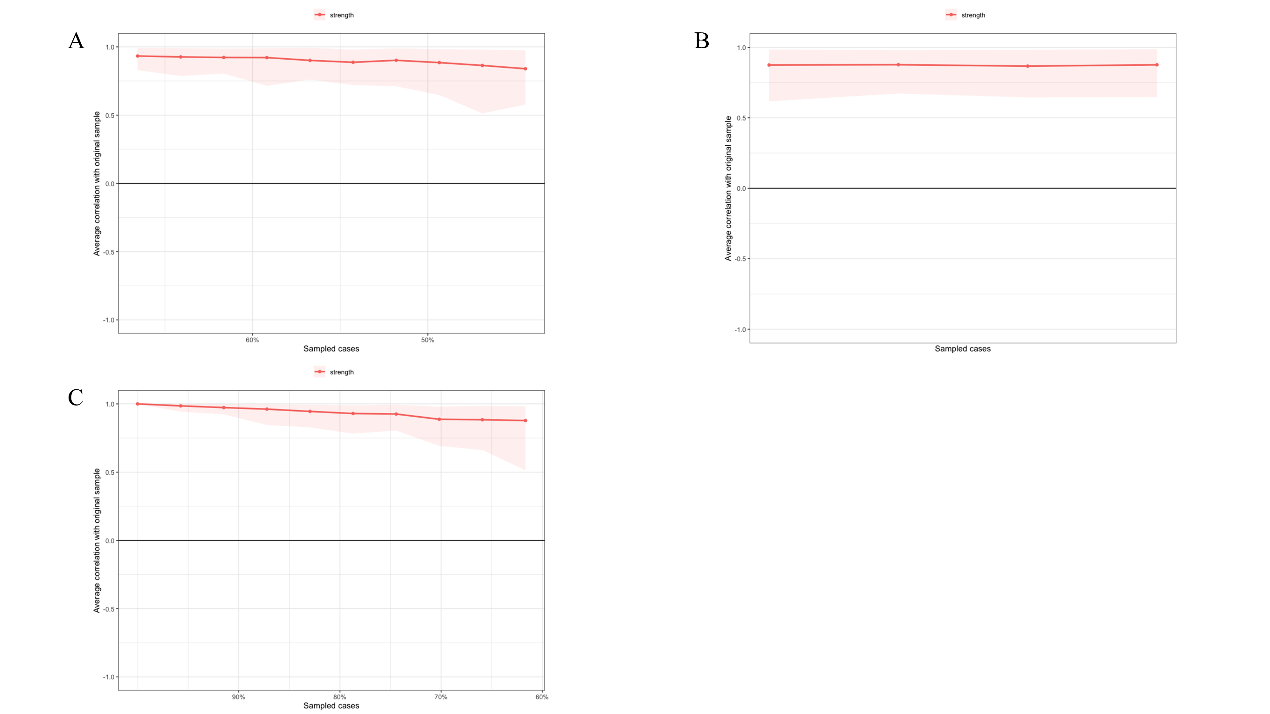


**Fig. S3.** **Stability of centrality indices by case dropping subset bootstrap.** The *x-*axis represents the percentage of cases of the original sample used at each step. The *y-*axis represents the average of correlations between the centrality indices from the original network and the centrality indices from the networks that were re-estimated after excluding increasing percentages of cases. Each line indicates that correlations among strength, A, elderly adults. B, male elderly. C, female elderly.


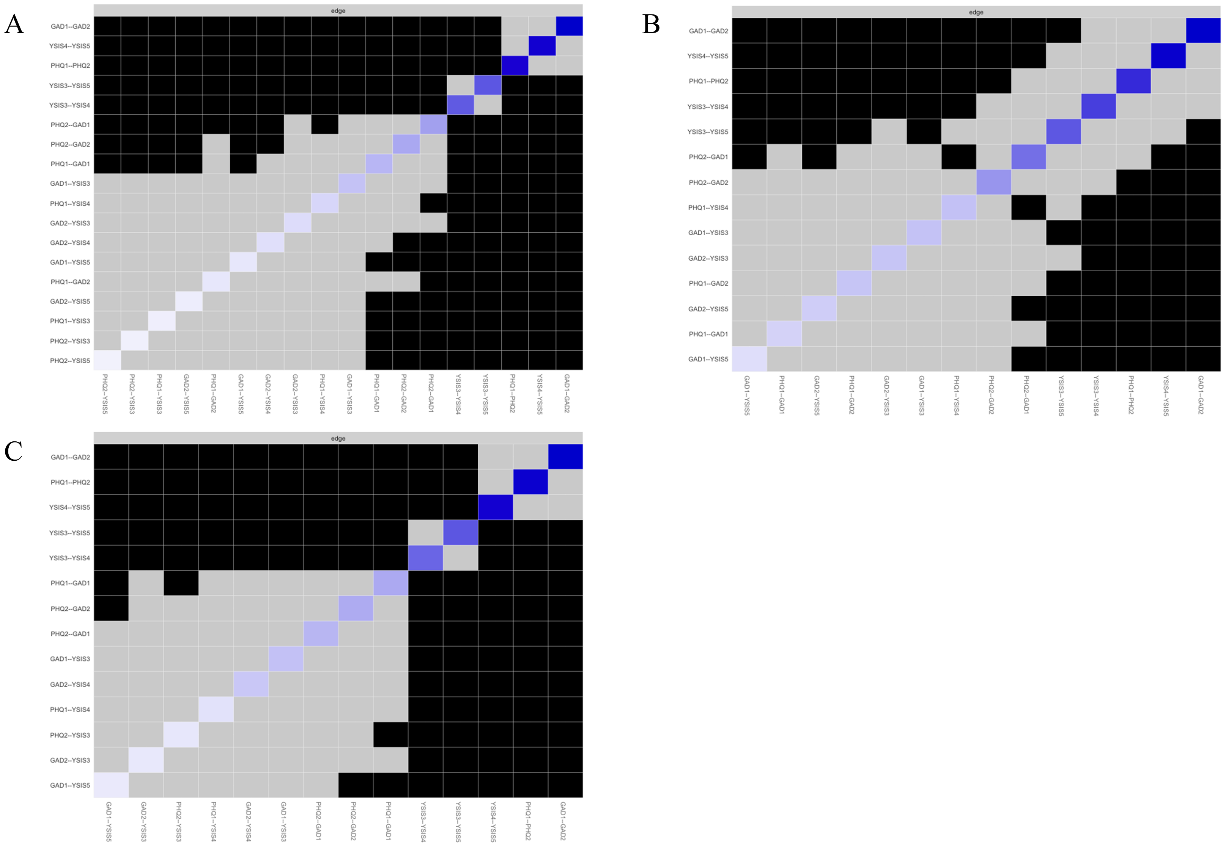


**Fig. S4.** Nonparametric bootstrapped difference test for edges. Grey boxes indicate no significant difference, whereas black boxes indicate a statistically significant difference (*p < .05*). Diagonal colour and saturation represent the magnitude and direction of each estimated edges, A, elderly adults. B, male elderly. C, female elderly.


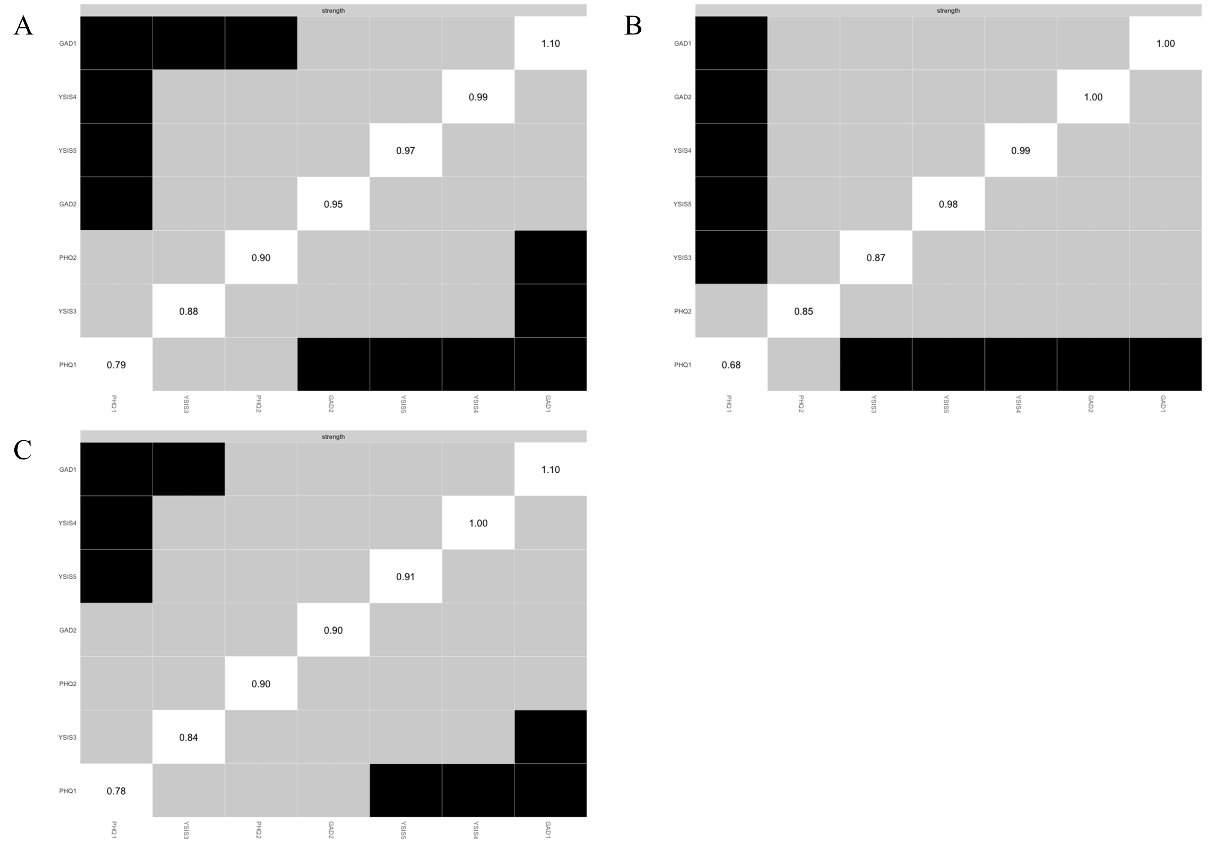


**Fig. S5.** Nonparametric bootstrapped difference test for nodes. Grey boxes indicate no significant difference, whereas black boxes indicate a statistically significant difference (*p < .05*). A, elderly adults. B, male elderly. C, female elderly.

**Table S4.** Means, standard deviations, *t*-test, *p-value*, and *Cohen's d* in males (n = 409) and females (n = 893).

|  | Male  *Mean (SD)* | Female  *Mean (SD)* | *p* | *Cohen’s d* |
| --- | --- | --- | --- | --- |
| PHQ1 | 0.53 (0.86) | 0.49 (0.83) | 0.49 | -0.04 |
| PHQ2 | 0.41 (0.75) | 0.41 (0.74) | 1.00 | 0 |
| GAD1 | 1.42 (0.73) | 1.39 (0.73) | 0.63 | -0.03 |
| GAD2 | 1.39 (0.73) | 1.37 (0.74) | 0.63 | -0.03 |
| Sleep3 | 1.67 (1.09) | 1.80 (1.14) | 0.05 | 0.11 |
| Sleep4 | 1.83 (1.20) | 1.90 (1.21) | 0.31 | 0.06 |
| Sleep5 | 1.75 (1.13) | 1.87 (1.22) | 0.10 | 0.10 |
